# Li-Fraumeni Syndrome

**Published:** 2017-11-01

**Authors:** Wendy H. Vogel

**Affiliations:** Wellmont Cancer Institute, Kingsport, Tennessee

Li-Fraumeni syndrome is a rare inherited autosomal-dominant disorder that is manifested by a wide range of malignancies that appear at an unusually early age. Li-Fraumeni syndrome is also known as sarcoma, breast, leukemia, and adrenal gland (SBLA) cancer syndrome. This syndrome first appeared in the medical literature around 1969 (Li & Fraumeni, 1969a, 1969b). Other malignancies associated with this syndrome include malignancies of the lung, pancreas, skin, gastrointestinal tract, choroid plexus, and germ cell, as well as lymphoma, early-onset colorectal cancer, and Wilms tumor (Agarwal et al., 2014). 

It is estimated that germline *TP53* mutations are responsible for around 1% of hereditary breast cancer (Daly et al., 2010; National Comprehensive Cancer Network [NCCN], 2017). There also appears to be an association between germline *TP53* mutations and human epidermal growth factor receptor 2 (HER2)–positive breast cancer (Melhem-Bertrandt et al., 2011; NCCN, 2017; Wilson et al., 2010). Li-Fraumeni syndrome is associated with abnormalities in the tumor protein p53 gene (*TP53*), located on chromosome 17p13.1. *TP53* is considered a tumor-suppressor gene with influences on cell-cycle arrest, apoptosis, and DNA repair (Mai et al., 2012). It is a common somatic mutation seen in most cancer types, but when there is a germline mutation, this causes Li-Fraumeni syndrome (Bouaoun et al., 2016).

Most of the deleterious germline mutations occur in DNA-binding regions (Mai et al., 2012). When there is a mutation of *TP53*, there is a loss of tumor suppression and a resulting gain of oncogenic function (Agarwal et al., 2014). *TP53* germline mutations are highly penetrant, with a cumulative lifetime incidence of cancer of almost 100% (NCCN, 2017). *TP53* mutations are present in about 80% of families with Li-Fraumeni syndrome (Andrade et al., 2016). There are families with Li-Fraumeni–like features but without this gene mutation, which is likely due to other mutated genes. Currently, it is believed that *TP53* mutations may be more common than previously thought, with estimates of 1 in 5,000 to 1 in 20,000 (NCCN, 2017). 

Cancers associated with Li-Fraumeni syndrome often occur at earlier-than-expected ages; for example, 80% of bone and soft-tissue sarcomas and breast cancer associated with the syndrome occur prior to age 45 (Yurgelun et al., 2015). For women, the lifetime risk of cancer approaches 90% to 100% by age 60. Men have an estimated lifetime risk of cancer of about 73%. In women, 50% will have a cancer by age 31 and in men, 50% will have a cancer by age 46 (Mai et al., 2016). In individuals with Li-Fraumeni syndrome, there is a much higher risk of developing a second malignancy as well (about 30%–57%; Hisada, Garber, Li, Fung, & Fraumeni, 1998; Schneider, Zelley, Nichols, & Garber, 2013). The risk for the development of a third malignancy is about 38% (Schneider et al., 2013). 

## SCREENING RECOMMENDATIONS

There are multiple criteria for testing for Li-Fraumeni syndrome. The NCCN recommends two criteria: the classic criteria and the 2015 Revised Chompret criteria (NCCN, 2017). The classic criteria include the combination of an individual diagnosed with sarcoma prior to age 45; a first-degree relative diagnosed with cancer prior to age 45; and an additional first- or second-degree relative in the same lineage with cancer diagnosed prior to age 45 or sarcoma at any age (see Table 1). If the individual is from a family with a known *TP53* mutation, testing is appropriate. The 2015 Revised Chompret criteria detail the types of cancer for which to assess and allows a slightly later age at diagnosis (Chompret et al., 2001; NCCN, 2017; Table 1). 

**Table 1:**
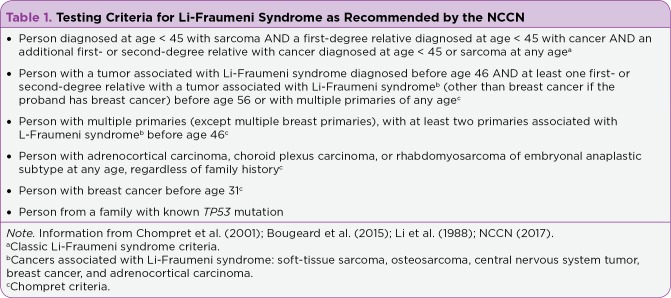
Testing Criteria for Li-Fraumeni Syndrome as Recommended by the NCCN

Screening for the detection of early cancer manifestation is key to prolonged survival in individuals with Li-Fraumeni syndrome. Screening should be directed to the personal medical history as well as to the specific pattern of cancer in the family (Schneider, Zelley, Nichols, & Garber, 2013). The Toronto Protocol is a screening protocol updated in 2016 for the follow-up of persons with Li-Fraumeni syndrome diagnosed with certain cancers (Villani et al., 2016).

The NCCN recommendations for screening are noted in Table 2. The NCCN recommends monthly breast self-examination (called "breast awareness"), clinical examination by a health-care provider twice a year, and annual imaging (mammography or magnetic resonance imaging [MRI]). In general, noninvasive screening should begin around age 18 to 20 years, and diagnostic imaging should begin at age 20 to 25 years. Screening for colorectal cancer would include the initiation of screening at an early age (25 years or 5 years prior to the earliest diagnosis in the family) and an increased frequency of screening (every 2 to 5 years; NCCN, 2017). Risks of cancer and limitations of screening should be discussed with the patient and with other health-care providers (NCCN, 2017). Ideally, screening should occur within the context of a clinical trial (Ballinger, Mitchell, & Thomas, 2015). 

**Table 2:**
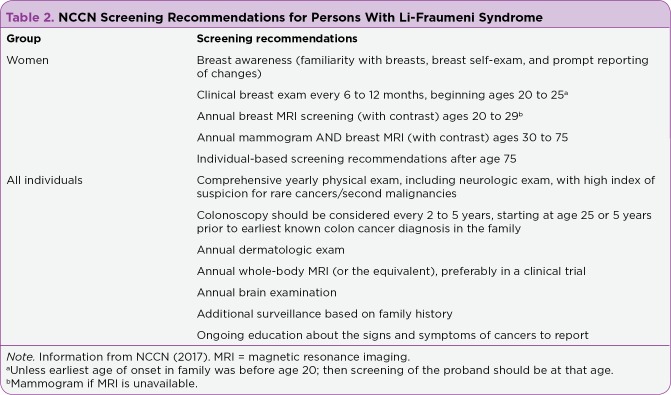
NCCN Screening Recommendations for Persons With Li-Fraumeni Syndrome

Annual whole-body MRI is recommended by the NCCN (2017) in individuals with Li-Fraumeni syndrome. This might be advantageous due to the risk of cumulative radiation exposure (Schneider et al., 2013). Clinical trials are ongoing internationally to assess the potential benefit of whole-body MRI (Ballinger et al., 2015). Several studies have demonstrated that an intensive screening program including whole-body MRI imaging can detect tumors early, thus improving long-term survival (Ballinger et al., 2015; Saya et al., 2017; Villani et al., 2016). Psychosocial issues may ensue due to frequent screening and should be addressed (McBride et al., 2017). Anxiety, depression, and grief are just a few psychosocial issues that an individual with a hereditary predisposition to cancer might experience.

Persons with a germline mutation of *TP53* should be followed closely with frequent screening exams as previously discussed. General follow-up measures include an annual physical examination, including careful skin and neurologic examinations (NCCN, 2017). Individuals should be counseled to seek medical attention for evaluation of any unexplained symptoms (see Table 3). 

**Table 3:**
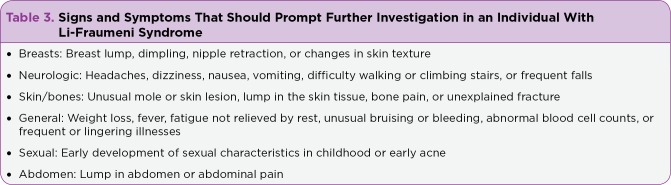
Signs and Symptoms That Should Prompt Further Investigation in an Individual With Li-Fraumeni Syndrome

## PREVENTIVE MEASURES

When possible, preventative measures should be considered. Prophylactic mastectomy may also be an option for some women (Schneider et al., 2013), although there are no clear data available on its efficacy (NCCN, 2017). Individuals should be counseled about behavioral methods of prevention, such as tobacco use cessation, weight control, healthy eating habits, regular exercise, moderate use of or avoidance of alcohol, and sun safety measures (Schneider et al., 2013). Prophylactic pharmacologic agents are under study, including metformin for the prevention of cancer (National Cancer Institute, 2016). Metformin has been associated with a reduced cancer risk in several epidemiologic studies (National Cancer Institute, 2016). 

## TREATMENT AND PATIENT EDUCATION

Treatment of cancer associated with Li-Fraumeni syndrome is generally no different from any other cancer treatment (Agarwal et al., 2014; Schneider et al., 2013). However, radiation-associated secondary cancers are more common in this population; therefore, radiation is avoided whenever possible unless the benefits of radiation outweigh its risks (Mirzayans, Andrais, Scott, Wang, & Murray, 2013; NCCN, 2017). This would mean that mastectomy is recommended over lumpectomy to avoid radiation and due to the increased risk of a second primary breast cancer (Schneider et al., 2013). To date, there are no specific drugs recommended to target the *TP53* mutation; however, clinical trials are addressing targeted treatments for various *TP53* mutations (Bouaoun et al., 2016). 

Persons with Li-Fraumeni syndrome require much education about this genetic disorder, the types of cancers they have an increased risk for, and the signs and symptoms of these cancers. Table 3 reviews these signs and symptoms. Screening recommendations should be shared with the patient, and any abnormalities should be addressed promptly. Reproductive options for those of childbearing age should be discussed. This could include prenatal diagnosis with preimplantation-assisted reproduction (NCCN, 2017). Psychosocial counseling may be indicated for distress related to the diagnosis and screening (Peters et al., 2016). Partners or significant others of the affected individual may also be at risk for psychosocial distress (Lammens et al., 2011). Resources available for persons with Li-Fraumeni syndrome are listed in Table 4.

**Table 4:**
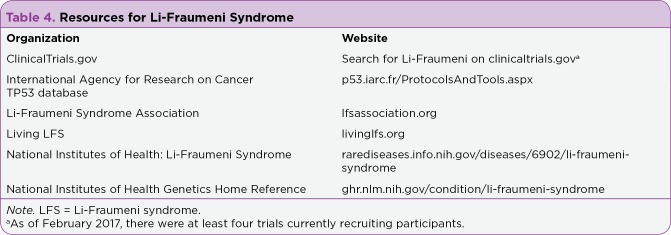
Resources for Li-Fraumeni Syndrome
